# Exosomal noncoding RNAs in central nervous system diseases: biological functions and potential clinical applications

**DOI:** 10.3389/fnmol.2022.1004221

**Published:** 2022-11-09

**Authors:** Zhong-Yu Wang, Zeng-Jin Wen, Hai-Ming Xu, Yu Zhang, Yin-Feng Zhang

**Affiliations:** ^1^Institute for Translational Medicine, The Affiliated Hospital of Qingdao University, College of Medicine, Qingdao University, Qingdao, China; ^2^Department of Occupational and Environmental Medicine, School of Public Health and Management, Ningxia Medical University, Yinchuan, China; ^3^The Key Laboratory of Environmental Factors and Chronic Disease Control of Ningxia, Ningxia Medical University, Yinchuan, China

**Keywords:** exosome, noncoding RNA, central nervous system, biomarkers, pathophysiology, therapy

## Abstract

Central nervous system (CNS) disease is a general term for a series of complex and diverse diseases, including Alzheimer’s disease (AD), Parkinson’s disease (PD), multiple sclerosis (MS), CNS tumors, stroke, epilepsy, and amyotrophic lateral sclerosis (ALS). Interneuron and neuron-glia cells communicate with each other through their homeostatic microenvironment. Exosomes in the microenvironment have crucial impacts on interneuron and neuron-glia cells by transferring their contents, such as proteins, lipids, and ncRNAs, constituting a novel form of cell-to-cell interaction and communication. Exosomal noncoding RNAs (ncRNAs), including microRNAs (miRNAs), long noncoding RNAs (lncRNAs), circular RNAs (circRNAs), and PIWI-interacting RNAs (piRNAs), regulate physiological functions and maintain CNS homeostasis. Exosomes are regarded as extracellular messengers that transfer ncRNAs between neurons and body fluids due to their ability to cross the blood-brain barrier. This review aims to summarize the current understanding of exosomal ncRNAs in CNS diseases, including prospective diagnostic biomarkers, pathological regulators, therapeutic strategies and clinical applications. We also provide an all-sided discussion of the comparison with some similar CNS diseases and the main limitations and challenges for exosomal ncRNAs in clinical applications.

## Introduction

Central nervous system (CNS) diseases are numerous and all-encompassing. They not only involve common neurodegeneration, such as Alzheimer’s disease (AD), Parkinson’s disease (PD), multiple sclerosis (MS), and amyotrophic lateral sclerosis (ALS) but also tumors, stroke and epilepsy, which seriously endanger the quality of life and safety of patients (Prince et al., 2013; Xia et al., 2019; Cheng et al., 2020; Pregnolato et al., 2021). Irreversible damage, cognitive impairment and treatment resistance, which occur in CNS disease, together with the poor understanding of molecular pathogenesis and the lack of timely diagnosis and sensitive therapeutic monitoring tools have largely impeded the available countermeasures and resulted in the terrible prognosis of CNS disease patients (Rastogi et al., 2021). Therefore, it is imperative to clarify the molecular mechanisms underlying CNS disease development and progression and foster the ever-increasing passion for the research of early diagnostics and new treatment modalities for CNS diseases.

The occurrence of the CNS diseases mentioned above is closely related to molecular changes in the microenvironment, which determine the specificity, heterogeneity and hallmark features of CNS diseases (Chivet et al., 2012). For instance, nerve cells convert cell phenotype and accumulate toxic substances by changes in the microenvironment, gradually leading to neuron loss and neuron degeneration (Tian et al., 2021). Moreover, emerging evidence has revealed that exosomes in the microenvironment released from neurons or glial cells have crucial impacts on both interneuron and neuron-glial cells, which constitutes a novel form of cell-to-cell interaction and communication (Tielking et al., 2019; Wang G. et al., 2020). Illuminating the mystery of the mechanism of intercellular communication in CNS cells with the microenvironment is conducive to exposing multiple potential therapeutic targets. Exosomes get through multiple stages, such as EE (early endosome), ILE (intraluminal vesicle), and MVBs (multivesicular bodies). Brain-derived exosomes have many unique features that penetrate blood-brain barrier (BBB) easily and can travel between neurons and various gliacytes *via* the cerebrospinal fluid (CSF) and extracellular space, which control CNS homeostasis or activate cytotoxic responses with recipient cells (Saugstad et al., 2017). This also suggests that the number, nature and composition of specific exosomes can be used to diagnose CNS diseases in their early stages (Shaimardanova et al., 2020). Furthermore, exosomes play a promising role in intercellular communication by transferring bioactive cargoes between spines of the same releasing neuron or to afferent neurons (Rufino-Ramos et al., 2017). Proteins, DNA, mRNA, lipids, and ncRNAs have been detected in exosomes that can also modulate gene expression in target cells and influence the hallmarks of neurons. ncRNAs are luxuriant and stable in exosomes, while this uncovers the feasibility of using exosomes as a new means of ncRNA carrier to the CNS and might also provide new diagnosis and prediction strategies to alleviate and reverse neurological disturbances of CNS diseases (Lizarraga-Valderrama and Sheridan, 2021). This review describes current research on the roles of exosomal ncRNAs in CNS diseases as well as their modern approaches to diagnosis and treatment.

## Exosomes Biogenesis and Characterization

**Figure 1:**
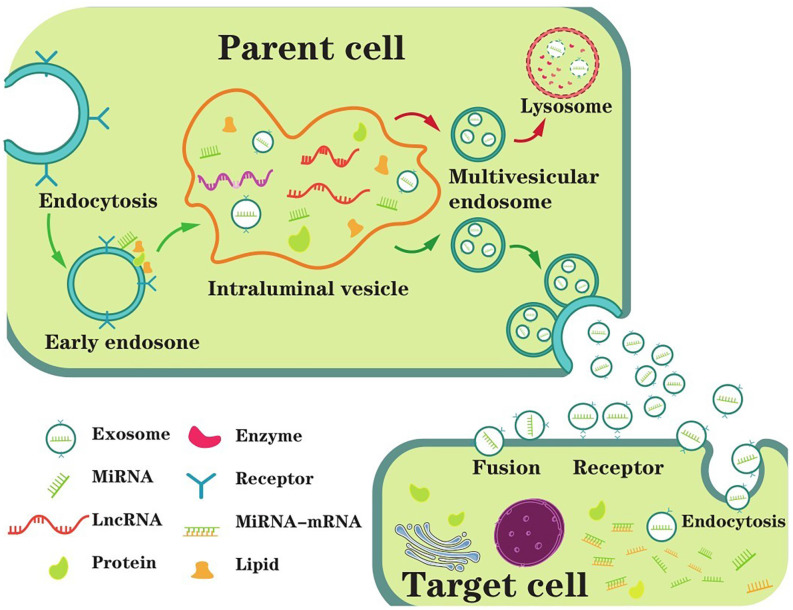
The main process of exosome biogenesis, secretion, and ingestion. The biogenesis of exosomes starts with the formation of EEs (early endosomes) through endocytosis at the plasma membrane. Then, the EE begins inward budding starting from the membrane to form the endosome lumen, sequestering ncRNAs and other cytoplasmic molecules and eventually converting to intraluminal vesicles (ILVs). MVBs (multivesicular bodies) are matured from the segregation of the lumen from the plasma membrane leading to molecule accumulation in the ILVs, which are also called late endosomes. Generally, MVBs either fuse with the plasma membrane or fuse with the lysosome for degradation. In extracellular space, exosomes are untaken by target cells, mediating by endocytosis, fusion or receptor interaction. As a result, exosome contents are taken into recipient cells and exert biological functions.

Extracellular vesicles (EVs) were first identified 40 years ago as reticulocytes and had been shown to exist in a variety of biological fluids since then (Colombo et al., 2014). Classically, there are three types of EVs: exosomes (40–100 nm in diameter), microvesicles, and apoptotic bodies (50–2,000 nm in diameter; Thompson et al., 2016; Mathieu et al., 2019; Xia et al., 2019). The latter two can be shed directly from the plasma membrane; however, exosomes need to be released upon fusion of MVBs with the plasma membrane (Coleman and Hill, 2015). More explicitly, exosomes originate from the endosome system, which mainly experienced three stages, including EE, MVBs, and intraluminal vesicles (ILVs; Lin and Shi, 2019). EEs can be formed by almost all types of cells, which represent the infant sorting compartment for internalized nucleic acids (DNA, ncRNAs, mRNAs, etc.) and other macromolecules in endocytic vesicles (Morelli et al., 2004; Scott et al., 2014). Soon afterward, the EEs begin inward budding starting from the periphery membrane to form the endosome lumen, sequestering cytoplasmic molecules or medicines and eventually converting to ILVs (Liu et al., 2019). In a stepwise manner, the segregation of the lumen from the plasma membrane leads to molecule accumulation within the ILVs, which mature into late endosomes, also called MVBs (van Niel et al., 2018). The fusion of MVBs with the plasma membrane was first observed in rat reticulocytes in 1983 (Harding et al., 1983); the same phenomenon was observed again in sheep reticulocytes in the same year (Pan and Johnstone, 1983). Some MVBs that have formed ILVs are directed to lysosomes, and their contents are degraded. In others, MVBs are transported to the plasma membrane and released to form exosomes. Because this fusion process resembles reverse endocytosis, which means releasing intracellular contents rather than internalizing external molecules, thus Rose Johnstone named them exosomes (Johnstone, 2005). In conclusion, exosomes are nanoscale vesicles released by the fusion of MVBs originating in the endocytic pathway with the plasma membrane (Figure 1).

Interest increased around these exosomes, as they appeared to be involved in many neuronic processes, such as neuron proliferation, differentiation, and apoptosis (Osier et al., 2018). For example, exosomes convey functional genetic information and proteins between cells, mediating intercellular communication between different cell types in the brain and thus affecting normal and pathological conditions (Colombo et al., 2014). Exosomes are also highly heterologous and can be detected in various human secretions and tissues of the CNS (He et al., 2018). As we mentioned above, exosomes participate in CNS disease biogenesis, and their contents are altered during disease, making them a particularly attractive target for novel diagnostic and therapeutic approaches (Malm et al., 2016).

### Exosome-derived noncoding RNAs (ncRNAs)

Noncoding RNAs (ncRNAs) in exosomes mainly include microRNAs (miRNAs), long noncoding RNAs (lncRNAs), circular RNAs (circRNAs), PIWI-interacting RNAs (piRNAs), and small interfering RNA (siRNAs), which play major roles in gene regulation (Budnik et al., 2016). With the further discovery and study of ncRNAs in exosomes, many innovative functions and applications have emerged, ranging from novel methods of cell-to-cell communication to promising disease biomarkers; and exosomal ncRNAs have new therapeutic applications given the indispensable functions of exosomes in the biogenesis of CNS diseases (Chen J. J. et al., 2017). In this review, we mainly focus on exosomal miRNAs, lncRNAs, circRNAs, and piRNAs in CNS.

The selection of these exosomal ncRNAs is regulated meticulously. Some of these exosomal ncRNAs are present in exosomes regardless of cellular origin, suggesting potential different mechanisms related to the sorting of ncRNA cargoes. For instance, miRNA to take shape ILVs relies on the endosomal sorting complex required for transport (ESCRT) proteins, and the neutral sphingomyelinase2 (nSMase2)-dependent pathway (Juan and Furthauer, 2018). These different kinds of ILVs containing ncRNAs are selectively packaged into different vesicles to form exosomes. Furthermore, several RNA-binding proteins (RBPs) motifs on some exosomal lncRNAs had been verified (Ahadi et al., 2016). The two most common motifs were associated with ELAVL1 and RBMX, resulting in a two-fold increase for these sites in exosomal lncRNAs when compared to cellular lncRNAs. This suggests that specific proteins could promote lncRNA to sort into exosomes, and lncRNA-RBP complexes might capture particular miRNAs and guide the packaging of them into exosomes. The selective mechanism of transporting ncRNAs to the exosome interior is a heterogenous process, as evidenced by their varied content, even in different types of CNS disease development. More detailed mechanisms of ncRNA selection into exosome need further exploration.

#### miRNA

miRNAs are single-stranded non-coding RNAs of 19–25 nucleotides with regulatory functions found in eukaryotes (Lu and Rothenberg, 2018). In the nucleus, the biogenesis of miRNA begins with the transcription of miRNA genes into large initial transcripts (pri-miRNA), which then produce a molecule of approximately 70 nucleotides with a stem-loop structure (pre-miRNA) under the shearing action of RNase III endonuclease Drosha and the RNA-binding protein DGCR8 (Brate et al., 2018; Dexheimer and Cochella, 2020). In the cytoplasm, pre-miRNAs are cleaved into mature miRNAs by the RNase III enzyme Dicer and then exported from the nucleus. With the help of helicase, mature miRNAs can shape the RNA-induced silencing complex (RISC), and complementary mRNA sites regulate gene expression *via* base pairing (Higa et al., 2014). As the smallest nucleic acid, exosome-derived miRNAs in the nervous system constitute a complex network for carrying genetic information and regulating gene expression, indicating that exosomal miRNAs play a key role in the regulation of the occurrence, development, and spread of many human CNS diseases (Lukiw and Pogue, 2020).

#### lncRNA

Although miRNA transcripts are small, some ncRNAs can surpass 200 nucleotides in length, and they are therefore cataloged as lncRNAs (Derrien et al., 2012; Wu and Kuo, 2020). lncRNAs can exist in both the nucleus and cytoplasm and play different functions according to their subcellular localization (Statello et al., 2021). In the nucleus, lncRNAs are involved in transcriptional regulation of gene expression and mRNA splicing. In the cytoplasm, such as in exosomes, they can affect the stability of mRNA and regulate the biological functions of proteins (Zhu et al., 2013). In addition, lncRNAs share some comparable features with mRNAs: they undergo posttranscriptional modifications, such as 5’-capping, polyadenylation, and alternative splicing (Ponting et al., 2009). The other way around, particularities of numerous lncRNAs are not found in mRNAs, such as cis-regulatory capacity, lacking robustly translated open reading frames, special 3’-terminal processing, templating of nucleic acid polymerization or assembly, and other divergences (Quinn and Chang, 2016). Concurrently, the growing ranks have motivated an increased focus on understanding the roles of exosomal lncRNAs in biology, steadily revealing that exosomal lncRNAs preside over the occurrence of many neurodegenerative diseases and the damage processes of the CNS (Szilagyi et al., 2020).

#### circRNA

circRNA is a naturally occurring class of ncRNA molecules that have become a current research focus in the field of RNA (Li et al., 2015). Unlike conventional linear RNA, circRNA has a covalently closed continuous loop and is resistant to RNA exonuclease, so its expression level is more stable, and it is not easily degraded (Qu et al., 2015). In fact, hundreds of circRNAs are enriched in human brain tissues and have distinctive regulatory potency (Rybak-Wolf et al., 2015). Recent research has discovered that exosomal circRNAs can function as miRNA sponges, protein inhibition, and regulators of splicing and transcription; and they also can bind RBP, as well as translate into peptides and proteins, which contribute to neurodegenerative diseases up to a point (Lukiw, 2013; Fanale et al., 2018; Welden et al., 2018). These rising studies indicate that such a circRNA-miRNA competing system is a non-negligible epigenetic regulatory aspect that controls gene expression in some CNS diseases.

Although research on exosomal circRNA is still at a premier period, an emerging number of studies suggest that circRNAs by virtue of exosomes institute their circulation and communicate with the recipient cells. Thus, carrying out exosomal circRNA abundant biological functions (Wang Y. et al., 2019).

#### piRNA

The piRNA that is 26–31 nucleotides in length is small non-coding RNA with 2’-O-methylation at their 3’ ends (Pippadpally and Venkatesh, 2020). In contrast to other ncRNAs, which are double-stranded derived RNAs that are dicer-dependent and bind to Argonaute proteins, piRNAs are transcribed from ssRNAs, dicer-independent and bound to PIWI proteins (Aravin et al., 2006). In contrast to miRNAs, piRNAs could silence genes, participate in various aspects, including transposon silencing, transcriptional silencing or activation, posttranscriptional regulation, and other modifications, further elucidating the uniqueness of piRNAs compared with other ncRNAs (Rajasethupathy et al., 2012; Ozata et al., 2019; Wang and Lin, 2021). piRNA biogenesis is unique and complex and is marked by the noncanonical transcription of precursor molecules and self-augmentation mechanisms. Similar to miRNAs and lncRNAs, piRNAs have become a contemporary research hotspot in the field of exosomal ncRNA and could be widely involved in the processes of life.

**Table 1:** The biological functions of exosomal ncRNAs in AD.

**Diseases**	**Classification of ncRNA**	**ncRNAs**	**Targets**	**Biological function**	**References**
Alzheimer’s disease	miRNA	miR-193b	APP	Propelling Aβ accumulation	Liu et al. (2014)
		miR-29c	APP	Affecting Aβ generation	Chen J. J. et al. (2019)
		miR-29a/b-1	BCAE1	Propelling APP expression and Aβ accumulation	Hébert et al. (2008)
		miR-125b	Tua, p44/42-MAPK	Promoting tau hyperphosphorylation	Banzhaf-Strathmann et al. (2014)
		miR-34a	RSN genes, oxidative phosphorylation proteins and glycolysis proteins	Dysfunction of synaptic plasticity, energy metabolism, and resting state network activity	Sarkar et al. (2016)
	lncRNA	BACE1-AS	BACE1	Propelling APP expression and Aβ accumulation	Wang D. et al. (2020)
		51A	SORL1	Impairing APP cleavage pathway and promoted neurotoxic Aβ formation	Andersen et al. (2005) and Cortini et al. (2019)
	circRNA	KIAA1586	miR-29b, miR-101, miR-15a	Acting as a ceRNA to disrupt the balance of three miRNA-related networks between miRNA and genes and accelerates APP accumulation	Zhang et al. (2019)
		ciRS-7	miRNA-7	Acting as a ceRNA to absorb miRNA-7 and decrease UBE2A expression	Lukiw (2013)
	piRNA	DQ597973 et.al	APOE	Participating in the changes of protein Tau and amyloid	Qiu et al. (2017) and Jain et al. (2019)

Interestingly, exosome might be a double-edged sword for ncRNAs (Wang Y. et al., 2019; Cheng et al., 2020). One is that exosome is protected by double-layer membrane wrapping that does help to secure the contents of exosomes from external interference and facilitate diffusion between neurons. Furthermore, in comparison with ncRNAs which exist outside exosomes, exosomal ncRNAs are protected from enzyme degradation and could be more easily delivered to produce targeted biological effects (Liu et al., 2022). On the other hand, exosome could reduce the accumulation of ncRNAs and help to clear ncRNAs which means ncRNAs could be taken up by other specific cells, such as macrophages.

The appearance of complicated diseases is the result of the synergism of multiple interacting genes or RNAs (Zhang et al., 2018). Hence, we should investigate disease mechanisms at the level of cell biology and unearth effective information from a vast network of interacting genes or RNAs.

## The Regulatory Functions of Exosomal NcRNAs in The Central Nervous System (CNS)

Exosomal ncRNAs in the microenvironment have crucial impacts on both interneuron and neuron-glial cells, which constitute a novel form of cell-to-cell interaction. Many promising studies have verified that exosomal ncRNAs could be vital regulatory factors in the pathogenesis of numerous CNS diseases (Li et al., 2018; Liu et al., 2019; Xia et al., 2019; Wu and Kuo, 2020). Here, we summarized recent findings related to the functions of exosomal ncRNAs in the pathogenesis of CNS diseases.

### Alzheimer’s disease (AD)

AD refers to a particular onset, course of cognitive and functional decline associated with particular neuropathology and age (Soria Lopez et al., 2019). Currently, although the primary causes of AD are still contentious, it is highly acknowledged that the accumulation of amyloid beta (Aβ), neurofibrillary tangles, synaptic loss, oxidative stress, and autophagy might play vital roles in AD neuropathology (Schneider and Mandelkow, 2008; Spires-Jones and Hyman, 2014; Lee et al., 2019). When amyloid precursor protein (APP) is cleaved, amyloidogenic Aβ fragments are produced, and then are mainly exported through exosomes. Then Aβ further clusters into amyloid plaques, which are considered to be responsible for the death of neurons (Weiner, 2013). Recent studies have shown that exosomal ncRNAs were also closely related to the biogenesis and progression of AD, suggesting that exosomal ncRNAs could participate in the pathogenetic process of AD (Li et al., 2018; Table 1).

#### The functions of exosomal miRNAs in AD

In 2017, Sarkar observed that the increased miR-34a expression in a specific brain region was related to the severity of AD pathology (Sarkar et al., 2016). They further found that miR-34a targeted dozens of many genes that might result in the dysfunction of synaptic plasticity, energy metabolism, and resting state network activity. Their findings implied that up-regulated miR-34a influenced neuron stability, which might be a potential mechanism of AD biogenesis. Furthermore, a research group demonstrated that miRNA-193b was a regulator of APP derived from blood and CSF (Liu et al., 2014). In samples from APP/PS1 double-transgenic mice, mild cognitive impairment (MCI) and dementia of Alzheimer-type (DAT) patients, this study found that miR-193b overexpression could repress the mRNA and APP protein expression that would influence Aβ generation in brain (Liu et al., 2014). Another interesting study reported that in primary neuronal culture and AD patients’ brain, the miR-29a/b-1 cluster was significantly downregulated, and β-site amyloid precursor protein cleaving the enzyme-1 (BACE1) expression was increased (Hébert et al., 2008). Soon afterward, Seongju Lee found that both miR-29a and miR-29b-1 could bind to the 3’ -UTR of BACE1, and the BACE1 levels were negatively correlated with the expression levels of these miRNAs (Lee et al., 2019). The integration of the information above reveals that miR-125-5p, miR-193b, and miR-29a/b1 could enter cells *via* exosomes, then influence Aβ production and ultimately lead to the amyloid plaque deposition. Above all, exosomal miRNAs are involved in many pathways that contribute to the biogenesis of AD, such as neuroplasticity, neuron network activity, accumulation of APP and Aβ, and expression, phosphorylation, and aggregation of tau.

#### The functions of exosomal lncRNAs in AD

In addition to miRNAs, many exosomal lncRNAs are also involved in the pathological process of AD. The expression of lncRNA BACE1-AS (β-site amyloid precursor protein cleaving the enzyme-1-antisense transcript) was significantly increased compared with that in healthy controls (Wang D. et al., 2020). BACE1-AS is the opposite strand to BACE1 and serves as a suppressor of the production of BACE1 (Luo and Chen, 2016). It is suggested that lncRNA BACE1-AS may affect the production of Aβ and participate in the pathology of AD. Moreover, multiple research groups demonstrated that the expression levels of lncRNAs 17A, 51A, and BC200 in plasma exosomes were elevated in certain brain regions in AD patients (Andersen et al., 2005; Cortini et al., 2019; Wang D. et al., 2020). Separately, 51A was found located on the antisense strand of the first intron of the sortilin-related receptor 1 (SORL1), impairing the APP cleavage and promoting neurotoxic Aβ formation (Weiner, 2013). Additionally, lncRNA17A might participate in neuroinflammation, and the binding between BC200 and eukaryotic initiation factor 4A (eIF4A; Ames et al., 2017; Guo et al., 2019). The function of exosomal lncRNA17A results in decoupling ATP hydrolysis from RNA duplex unwinding that preserved long-term neuronal plasticity. All of these studies indicated that BACE1-AS, 51A, 17A, and BC200 might serve as direct or indirect roles in AD pathogenesis and novel therapeutic strategies.

#### The functions of exosomal circRNAs in AD

In addition to miRNAs and lncRNAs discussed above, circRNAs may also play a role in the balance of miRNA networks; and the function of exosomal circRNA contributes to Aβ fibrils, oxidative stress, and progressive cognitive deficits, which is regarded as one potential mechanism of AD. For example, circRNA for miRNA-7 (ciRS-7) competes with anti-complementary miRNA complementary content and adsorbs miRNA-7. Hence ciRS-7 quenches the normal function of miRNA-7 and plays an important post-transcriptional regulator of human brain gene expression (Lukiw, 2013). Zhang et al. (2019) further found that circRNA KIAA1586 occurred frequently in AD risk ncRNAs. As a competing endogenous circRNA, its function was to competitively bind three known AD-risk miRNAs (miR-29b, miR-15a, and miR-101) to silence target genes and display the over-expression of Aβ peptide. As mentioned above, exosomal circRNAs serve as the pathological mediators of AD, which is a rapidly growing field of potential biomarkers, though more detailed studies are needed.

**Table 2:** The biological functions of exosomal ncRNAs in Parkinson’s diseases.

**Diseases**	**Classification of ncRNA**	**ncRNAs**	**Targets**	**Biological function**	**References**
Parkinson disease	miRNA	miR-153	α-syn	Mediating oxidative stress to influence α-synuclein levels	Gui et al. (2015)
		miR-325	ARC	Mediating inability to repress autophagic program	Bo et al. (2014) and Harischandra et al. (2018)
		miR-34a-5p	SYT-1, STX1A	Regulating different aspects of neurogenesis and synaptogenesis	Grossi et al. (2021)
		miR-137	OXR1	Induce oxidative stress damage in dopaminergic neurons	Jiang et al. (2019)
		miR-15b-5p, miR-30c-2-3p	KEGG	Affecting the expression of proteins encoded by target genes in dopaminergic synapse	Xie et al. (2020)
		miR-19b	PARK2, PARK8	Lossing of E3 ubiquitin-ligase activity and gaining of kinase activity	Heman-Ackah et al. (2013) and Cao et al. (2017)
	lncRNA	AK127687	LRRK2 mRNA	Inducing the stability of LRRK2 mRNA up-regulated and dopaminergic neuronal apoptosis	Elkouris et al. (2019)
		POU3F3	GCase, α-syn	Inducing upregulated exo/total α-syn ratio and decreased GCase activity	Zou et al. (2020)
		lnc-MKRN2-42:1	EIF4E, BTD, MKNK1, METTL5	Dysregulating neuronal apoptosis, synaptic remodeling, immunity and glutamate neurotransmitter metabolism	Wang Q. et al. (2020)

#### The functions of exosomal piRNAs in AD

Except for the many mechanisms that ncRNAs participate in, one vital pathological characteristic of AD is the stability of genes, which affect the expression of key proteins. Some studies had found that piRNAs take part in gene stability. The function of piRNAs is to silence repetitive genomic regions to mediate genomic stability; and some piRNAs were believed to play an active role in gene expression control, which was related to long-term memory (Landry et al., 2013). In 2019, Jain et al. (2019) first reported that piRNAs are differentially expressed in human CSF exosomes of AD dementia patients. Moreover, exosomal piRNAs might contribute to the pathogenesis of neuropsychiatric illnesses. For AD, piRNAs might influence the expression of APOE in the brain (a key protein-coding gene in AD), suggesting that piRNAs might be involved in the etiological processes of AD (Qiu et al., 2017). Jain demonstrated that piRNAs were significantly correlated with Tau protein levels or the Aβ42/40 ratio, indicating that these piRNAs could reflect changes in Tau protein or amyloid pathology (Jain et al., 2019).

In short, these exosomal ncRNAs have the same distinctive characteristics to participate in AD pathogenesis. Examples include APP and Aβ generation in the nervous system, consecutive hyperphosphorylation of tau, synaptic loss, oxidative stress, and neuronal death (Lee et al., 2019). In conclusion, these relevant studies reveal multiple functions of exosomal ncRNAs in the pathological process of AD and illustrate novel therapeutic targets for AD treatment.

### Parkinson’s disease (PD)

PD is the second most common neurodegenerative disease after AD and is considered a multifactorial disorder (Elbaz et al., 2016). Additionally, PD affects 1–2 per 1,000 of the population at various times and invades 1% of the population above the age of 60 years, and PD prevalence is increasing at different rates varying with age (Tysnes and Storstein, 2017). Furthermore, the pathology of PD is characterized by the progressive loss of dopaminergic neurons in the nigra pars compacta within the midbrain, accumulation of alpha-synuclein (α-SYN) into Lewy bodies and neurites and excessive neuroinflammation (Leggio et al., 2017). To date, it is clear that exosomal ncRNAs participate in neuron complex normal functions, differentiation, and apoptosis, which are regarded as neoteric mechanisms in the pathogenesis of PD (Table 2).

#### The functions of exosomal miRNAs in PD

Studies had shown that miRNAs in serum were encapsulated within exosomes, which were secreted by pathological tissues and quite stable (Andersen et al., 2014). More specifically, it had been documented that exosomal miRNAs played a significant role in the regulation of the list of PD-related pathogenic proteins, such as α-SYN, leucine-rich repeat kinase, Parkin, DJ-1/PARK7, and phosphatase (Gui et al., 2015). These biological proteins regulated many key pathways, including protein aggregation, autophagy, inflammation, and hypoxia (Harischandra et al., 2018). In 2019, a research group found that exosomal miR-137 was upregulated and played a vital role in the induction of oxidative stress injury in neurons (Jiang et al., 2019). Jiang further elaborated that miR-137 was found to directly target oxidation resistance 1 (OXR1) and negatively regulate its expression, thus inducing oxidative stress damage in dopaminergic neurons (Billia et al., 2013). The DJ-1 protein is encoded by the PARK7 gene and represents an important protein involved in autosomal recessive primary PD. Chen found that upregulated expression of miR-4639-5p in PD patients, which negatively regulated the post-transcription level of the DJ-1 gene and eventually caused severe oxidative stress and neuronal death (Chen et al., 2017a). The information mentioned above implies that exosomal miR-137-mediated and miR-4639-5p-induced oxidative stress may serve as a potential mechanism in PD pathology. Moreover, miR-325 was shown to be a complementary fragment with a caspase recruit domain to suppress the apoptosis repressor (an anti-autophagic protein), and thus could promote the autophagic cascade (Bo et al., 2014). Coincidentally, a study discovered that Mn exposure significantly increased miR-325 in exosomes, which suggested that exosomal miR-325 participated in dopaminergic neuron autophagy (Harischandra et al., 2018). Although these exosomal miRNAs were found to be able to target genes involved in many vital pathways for PD, some doubts and inexplicable puzzles remain. For instance, Doxakis found that miR-153 directly interacted with the specified regions of the α-SYN gene to inhibit translation from the chimeric transcript, and their effect was cumulative (Doxakis, 2010). Subsequently, a fascinating study reported that the expression of miR-153 was significantly up-regulated in exosomes from the CSF of PD patients compared with healthy controls (Gui et al., 2015). These self-contradictory reports demonstrated the complexity of PD and the immaturity of current techniques. In a word, these exosomal miRNAs can promote our understanding of the mechanisms of PD etiopathology and development. Although, these researches may facilitate the development of new strategies for the diagnosis and therapy of PD, they remind us that more specific studies are needed.

#### The functions of exosomal lncRNAs in PD

In addition to the classic exosomal miRNAs discussed above, many lncRNAs derived from exosomes are also involved in PD pathogenesis. In 2020, Zou et al. (2020) reported that exosomal linc-POU3F3 activity might shed light on the autophagic-lysosomal system in the pathogenesis of PD. They further applied the lncRNA microarray to detect the expression levels of various exosomal lncRNAs. Eventually, the difference in exosomal lncRNA expression was confirmed, and linc-POU3F3 presented the highest fold change value and the most stable detection density and it was therefore selected as the candidate lncRNA in the pathophysiological process of PD. In the same year, exosomal lnc-MKRN2-42:1 was verified to be related to the pathophysiology of PD by Wang Q. et al. (2020). Through bioinformatics analyses, they concluded that lnc-MKRN2-42:1 could regulate target genes such as EIF4E, BTD, MKNK1, and METTL5 and participated in functions, which correlated with apoptosis, synaptic remodeling, long-term potential, immunity, and glutamate neurotransmitter metabolism. Moreover, Elkouris showed that a mass of lncRNA genes was near to PD-linked protein-coding genes, and four of them were located in exosomes derived from human CSF (Elkouris et al., 2019). They demonstrated that exosomal lncRNAs participated in the direct differentiation of human iPSCs to dopaminergic neurons. Collectively, their data suggested that exosomal lncRNAs were associated with PD pathogenesis.

On the one hand, exosomes may act as vectors to facilitate ncRNA transmission between neurons. Hence, exosomal ncRNA takes part in PD biogenesis and is an emerging therapeutic target. However, further studies will be needed to investigate whether the expression levels of these exosomal ncRNAs would reflect their total brain levels and their more detailed and specific functional associations with PD pathology.

### Central Nervous System (CNS) tumors

**Figure 2:**
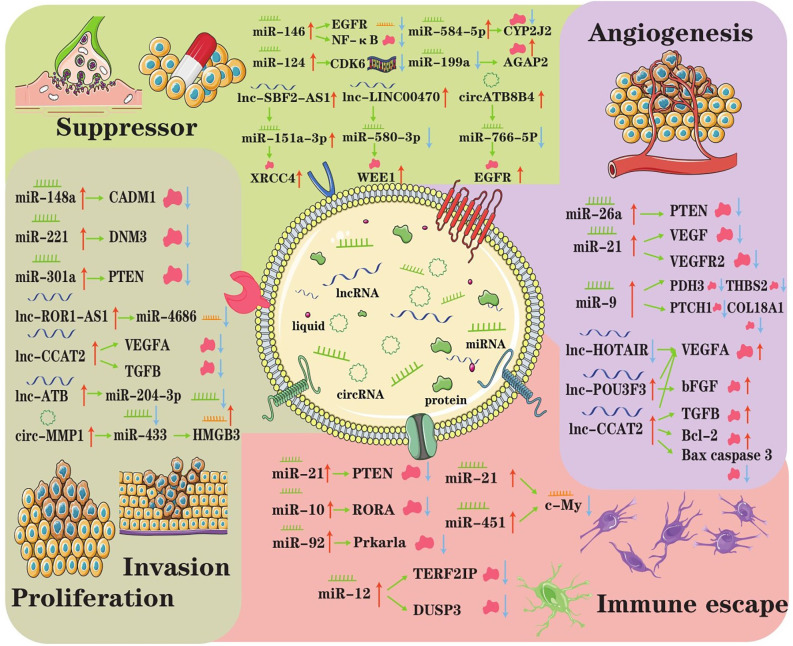
The underlying regulatory axes and molecular mechanisms of exosomal ncRNAs in CNS tumor. In the tumor microenvironment, intercellular communication can be achieved by exosomes. Subsequently, exosomal ncRNAs exert important roles in modulating treatment resistance, proliferation/invasion, angiogenesis, and immune escape.

When considering tissue and cell type, there are various kinds of CNS tumors, such as astrocytoma, oligodendroglioma, ependymoma, and medulloblastoma. Regardless of the types of tumors, they have common characteristics, such as rapid proliferation, extensive invasion, treatment resistance, angiogenesis, and immune escape. Burgeoning evidence indicates that exosomes mediate CNS tumor origination and progression by transporting specific biofunction molecules between different cell populations (Rooj et al., 2016). Accumulating studies have found that exosomes represented a new means of intercellular communication by delivering various bioactive molecules, such as ncRNAs and participate in tumor initiation and progression (Cheng et al., 2020). Here, we reviewed the present research on the roles of exosomal ncRNAs in overall stages of CNS tumor progression (Figure 2).

#### Exosomal ncRNAs and immune escape

Guo and colleagues found that hypoxia-induced exosomal miR-29a and miR-92a expression leads to the propagation of MDSCs (myeloid-derived suppressor cells), which play vital roles in mediating the formation of an immunosuppressive environment and helping CNS tumors escape the host immune response (Guo et al., 2019). Mechanistically, miR-29a and miR-92a activate the proliferation and function of MDSCs by targeting high-mobility group box transcription factor 1 (Hbp1) and protein kinase cAMP-dependent type I regulatory subunit alpha (Prkar1a), respectively. Altogether, the results of the Guo study provided new insights into the role of glioma exosomal miRNAs in mediating the formation of immunosuppressive microenvironments in tumors and elucidated the underlying exosomal miR-29a/miR-92a-based regulatory mechanism.

#### Exosomal ncRNAs contribute to angiogenesis

Tumorigenesis significantly intensifies the intratumor microvessel density, and angiogenesis is critical for tumor growth, migration and invasion (Ames et al., 2017). Exosomal ncRNAs can have various effects on angiogenesis. First, miR-9 could be secreted from glioma cells *via* exosomes and was frequently upregulated in glioma specimens (Chen X. et al., 2019). Exosomal miR-9 could significantly enhance the proliferation, migration and invasion of glioma cells, promoting tumorigenesis and an increase in angiogenesis by targeting many malignant genes in glioma cells. Second, the levels of lnc-POU3F3 were upregulated in glioma tissue and significantly correlated with the advanced tumor stage (Lang et al., 2017). That study explored the mechanism by which glioma-derived exosomes affect angiogenesis in more detail. They both used A172-Exos and shA172-Exos to assay the ability of HBMECs human brain microvascular endothelial cells (HBMECs), while HBMECs rapidly internalized A172-Exos and shA172-Exos, and the expression level of linc-POU3F3 in A172-Exos was significantly higher than that in shA172Exos. Furthermore, the gene and protein expression levels of bFGF, bFGFR, VEGFA, and Angio in HBMECs treated with A172-Exos were much higher than those in HBMECs treated with shA172-Exos. These results indicated that gliomas could induce angiogenesis by secreting exosomes enriched in lnc-POU3F3. Exosomal miR-9 and lncRNA-POU3F3 function as promoters of angiogenesis, which is pivotal for glioma pathogenesis and a promising therapeutic target in glioma.

#### Exosomal ncRNAs and cell proliferation, invasion and metastasis in glioma

It has been documented that ectopic proliferation is the most basic characteristic of tumors, while invasion and metastasis are the most striking features of tumors (Cheng et al., 2020). Multiple research groups manifested that the expression of exosomal ncRNA is abnormal and that ncRNA has an important impact on tumor proliferation, invasion, and metastasis. In 2015, Zhang found that primary tumor cells with normal expression of PTEN (an important tumor suppressor) but lost PTEN expression after dissemination to the brain (Zhang et al., 2015). Interestingly, brain microenvironment-dependent, reversible PTEN mRNA and protein down-regulation are epigenetically regulated by exosomal miRNAs from astrocytes. Mechanistically, astrocyte-derived exosomes mediated the intercellular transfer of PTEN-targeting miRNAs, leading to increased secretion of cytokine chemokine (C-C motif) ligand 2 (CCL2), which reciprocally enhanced the outgrowth of brain metastatic tumor cells *via* promoting proliferation and reducing apoptosis. Thus, it can be seen that exosomal miRNA induced PTEN loss, which contributed to primary brain metastasis outgrowth.

In addition to miRNAs, exosomal lncRNAs also unwilling to lag behind that also play a role in the pathogenesis of CNS tumors. In glioma tissues, lncRNA ROR1-AS1 was upregulated and packaged into exosomes derived from tumor cells (Chai et al., 2020). Functional analysis showed that it acted as a sponge of miR-4686 and inhibited its expression to bring about the promotion of glioma development. It is not hard to see exosomal lncRNA ROR1-AS1 derived from tumor cells promoted glioma progression *via* the miR-4686 axis, and the high expression of ROR1AS1 indicated a poor prognosis in glioma patients. Another type of exosomal ncRNA, circRNA, can also promote glioma progression. Exosomal circRNA 0001445 was taken up and upregulated in glioma cells treated with exosomes (Jitsukawa et al., 2017). In addition, exosomal circRNA 0001445 acted as a sponge for miRNA-127-5p to upregulate the expression of sorting nexin 5 (SNX5), which is a critical regulator in cancers (Han et al., 2021). Taking the information mentioned above, these studies provide an original understanding of the molecular biogenesis of CNS tumor progression, suggesting a participant and therapeutic target role of exosomal ncRNA in CNS tumor patients.

#### Exosomal ncRNA as a suppressor

Exosomal ncRNA not only has a beneficial influence on tumor proliferation, invasion, and metastasis but also acts as an inhibitor for drug resistance, tumor progression and invasion (Zhou et al., 2022); from the latter aspect, making exosomal ncRNA is a promising therapeutic tool for CNS tumors. For example, Yao recently selected miR-15a and miR-92a as candidates for further studies and confirmed that they were underexpressed in M2 macrophage exosomes (Yao et al., 2021). The results of target gene validation revealed that miR-15a and miR-92a were bound to CCND1 and RAP1B, respectively. Thus, interference with the expression of CCND1 or RAP1B reduced the phosphorylation levels of AKT and mTOR, indicating that both CCND1 and RAP1B could activate the PI3K/AKT/mTOR signaling pathway. Another study found that mesenchymal stem cell-derived exosomal miRNA-133b suppressed glioma progression *via* the Wnt/β-catenin signaling pathway by targeting EZH2 (Xu et al., 2019). More detailed particulars are that MSC-derived exosomal miR-133b was found to target and negatively regulate EZH2 expression, and EZH2 silencing resulted in inhibited glioma cell proliferation, invasion, and migration. Therefore, these existing results suggested that exosomal miR-133b could attenuate glioma development by disrupting the Wnt/β-catenin signaling pathway and inhibiting EZH2. Similarly, Yue et al. (2019) showed that the Wnt/β-catenin pathway was also triggered by exosomal miR-301a, which resulted in the suppression of TCEAL7 (tumor suppressor); thus, exosomal miR-301a contributed to glioblastoma resistance to radiotherapy.

These studies unveiled that exosome is an important carrier for tumor cell communication and that exosomal ncRNAs play suppressor roles in CNS tumor and are potential biomarkers and therapeutic targets in different types of cancer.

Before-mentioned studies summarized the exosomal ncRNAs that have been implicated in the pathogenesis, immune escape, angiogenesis, and treatment of CNS tumors. These intriguing findings suggest that we must broaden our horizons to identify molecular mechanisms and increase our knowledge in the field of CNS tumor pathogenesis.

### Multiple Sclerosis (MS)

Multiple Sclerosis (MS) is a chronic autoimmune disease in the CNS. MS mostly affects people aged 20–50 years. Thus, MS is the main cause of nontraumatic neurological disability in young adults (Martinez and Peplow, 2020). Pathways of MS pathogenesis, such as neurotrophin, focal adhesion, and T-cell receptor signaling pathways, all participate in MS biogenesis on different levels (Ebrahimkhani et al., 2017). The imbalance of regulatory T cells (Treg), neuronal, and inflammatory T cells contribute to oligodendrocyte loss, demyelination, and failure to remyelinate damaged brain areas (Pusic et al., 2014; Table 3).

**Table 3:** The biological functions of exosomal ncRNAs in MS, stroke, epilepsy, ASL, and depression.

**Diseases**	**Classification of ncRNA**	**ncRNAs**	**Targets**	**Biological function**	**Reference**
Multiple sclerosis	miRNA	miR-326	Ets-1, CD47	Inducing TH17 differentiation and maturation in the immunopathogenesis	Azimi et al. (2019)
		Let-7i	IGF1R, TGFBR1	Decreasing IGF1R/TGFBR1 expression on CD4+ T cells	Kimura et al. (2018)
		miR-229	MBP	Inducing myelination and oxidative tolerance	Pusic et al. (2016)
Stroke	miRNA	miR-126	BDNF/TrkB/Akt	Mediating the decrease of infarct volume and cell apoptosis and increase of microvessel density	Wang J. et al. (2020)
			TNF-α, IL-1β	Mediates the inhibition of neuroinflammation	
		miR-17-92	PTEN	Mediating the increase of neural plasticity and functional recovery after stroke	Xin et al. (2017)
		miR-134	IL-6, hs-CRP	Mediating the intercellular brain injury in ischemic stroke	Zhou et al. (2018)
Epilepsy	miRNA	miR-346	GABRA5	Inducing depression of inhibitory neurotransmission	Gitai et al. (2020)
		miR-331-3p	mTOR	Inducing the abnormal electrical brain activity	
Amyotrophic lateral sclerosis	miRNA	miR-155	P2ry12, Egr1, Csf1r	Suppressing microglia phagocytosis functions and lead to neuroinflammation	Vaz et al. (2019)
		miR-146	TNF-α, IL-1β, HMGB1	Inducing inflammation in ALS	
		miR-494-3p	SEMA3A	Mediating the decrease of motor neuron survival *in vitro*	Varcianna et al. (2019)
Depression	miRNA	miR-9-5p	IL-1β, IL-6 and (TNF-α)	Promoting M1 polarization in microglia and exacerbating depression symptom	Xian et al. (2022)

#### The functions of exosomal miRNAs in MS

In 2018, Kimura found that the expression of transfection of exosomal miRNA-let-7i was upregulated and let-7i could inhibit the production of Treg cells, and the effect of MS exosomes was disabled when T cells were incubated with let-7i inhibitor before culture (Kimura et al., 2018). Further analysis revealed that exosomal miRNA-let-7i decreased the expression of insulin-like growth factor 1 receptor (IGF1R) and transformed growth factor β receptor 1 (TGFBR1) on immature CD4+ T cells and then inhibited their differentiation into Treg cells. Compared with healthy controls, in relapsing-remitting MS (RRMS) patients, significantly increased expression of miR-326 in exosomes was observed (Azimi et al., 2019). Junker et al. showed that miR-326 targets CD47 in brain resident cells. In turn, the CD47 molecule inhibits the phagocytic activity of macrophages, consequently reducing its expression, which could increase the degradation of myelin. Similarly, Du and colleagues expounded that miR-326, by targeting Ets-1, a negative regulator of Th17 differentiation, could induce the differentiation of mature T cells into Th17 cells, thereby increasing the severity of MS (Du et al., 2009). Because exosomal miR-326 contributes to the pathogenesis of MS, it is possible that inhibition of miR-326 expression in T-cell-derived exosomes or engineering them to carry selected miRNAs may be considered a promising therapeutic approach for the treatment of MS and may also be a potential clinical target in the course of MS (Azimi et al., 2019). Moreover, gray matter demyelination is increasingly recognized as an important component of MS (Geurts et al., 2007). Subsequently, Pusic found that serum-derived exosomes of young and environmentally enriched patients had high levels of miR-219 (Pusic and Kraig, 2014). They further confirmed that exosomal miR-219 was essential and sufficient for myelinating oligodendrocyte production by depressing the expression of inhibitory regulators of differentiation. As a further confirmation of miR-219 function, they inhibited miR-219 in young serum-derived exosomes and found that the protein transcript levels of these miR-219 target mRNAs decreased, resulting in these altered exosomes no longer increasing myelination in slice culture. In conclusion, exosomal miRNAs have been verified to regulate both immune responses and myelination, making them attractive candidates both for pharmacological intervention and as disease biomarkers (Mycko and Baranzini, 2020).

#### The functions of other ncRNAs in MS

Emerging evidence has indicated the potential role of ncRNAs in the regulation of gene expression of MS pathogenesis, which provides new opportunities to better understand the course of MS (Yang X. et al., 2018). For instance, lncRNA GAS5 was found to suppress the transcription of TRF4, a key factor controlling M2 polarization, which is a pivotal feature of MS pathogenesis (Sun et al., 2017). Another group discovered that circRNA affected the protein coding transcripts, which elucidated a pathway directly linked with STAT3, a critical factor for the inflammatory demyelination and polarization of the immune response toward Th17 (Zurawska et al., 2019). There have been some reports on the role of ncRNAs in the pathological process of MS, but the association between ncRNAs and exosomes and the effects of their interaction on MS remain to be studied.

### Stroke

The second outstanding cause of mortality worldwide is stroke, which contributes to a considerable burden on families, communities, and society (Feigin et al., 2014). Stroke is an acute neurologic disorder that can threaten life if left untreated for a certain amount of time or diagnosed late (Jafarzadeh-Esfehani et al., 2020). The classic risk factors for stroke include high blood pressure, cardiac diseases, intemperance, smoking, diabetes mellitus, lipid metabolism disorder, and obesity. Symptoms of stroke include disbalance with speech, numbness, vision impairment, severe headache, obvious weakness and stiffness throughout the body, and walking difficulty (Xia et al., 2019; Table 3).

In terms of the pathology of stroke, the common reason is blockage or rupture of the cerebral artery, which leads to ischemic and hemorrhagic stroke, respectively, and results in stroke becoming the most common cerebrovascular disease (Wang Z. et al., 2019). Importantly, among different types of strokes, ischemic stroke accounts for approximately 85% of all strokes (Mirzaei et al., 2017). Moreover, various studies have shown that exosomal ncRNAs play key roles in stroke pathogenesis, complications, and outcomes (Mirzaei et al., 2017; Li et al., 2021). For instance, Zhou with his colleagues isolated exosomes from the blood of stroke patients and found that exosomal miR-134 was highly expressed in stroke patients compared with the control group (Zhou et al., 2018). Further, they found that the level of serum exosomal miR-134 was positively correlated with the expression of IL-6 and hs-CRP, which were both reported to reflect the degree of brain ischemic damage and stroke (Waje-Andreassen et al., 2005; Chaudhuri et al., 2013; Zhou et al., 2018). This study suggests that the enhanced production of exosomal miR-134 in stroke patients might induce intercellular brain injury *via* IL-6 and other cytokines. Except for the direct factors that contribute to the occurrence of stroke, numerous mechanisms influence stroke biogenesis, including angiogenesis, neurovascular integrity, inflammation, and synaptic plasticity. Recent findings showed that miR-134 regulated ischemia injury-induced neuronal cell death, indicating that exosomal miR-134 might also participate in the pathology of stroke by promoting neuroinflammation and neuronal death (Huang et al., 2015).

Abundant studies have demonstrated that the expression levels of many exosomal ncRNAs have an apparent difference compared with healthy controls, however, by which exosomal ncRNAs regulate the pathogenesis of stroke remains unknown. For example, miR-124 mediated an increase in angiogenesis and offered considerable neuroprotection against stroke, showing the neurorestorative and neuroprotective potential of miR-124 (Doeppner et al., 2013). However, the expression of miR-124 in stroke patients has not been detected as downregulated. These inspiring studies hint to us that a more incisive understanding of the role of exosomal ncRNAs in stroke pathogenesis needs to be established and could contribute to the discovery and development of new therapeutic approaches for stroke.

### Epilepsy

Epilepsy is one of the most common serious brain conditions, affecting over 70 million people worldwide (Thijs et al., 2019). In the nursing and older age groups, the incidence of epilepsy has a bimodal distribution with the highest stake. Epilepsy is characterized by intermittent and reduplicative spontaneous epileptic seizure with a verity of neurobiological, cognitive, psychological, and social consequences (Fisher et al., 2014). Although epilepsy results in tremendous passive impacts on people and society, the mechanisms of its pathogenesis are poorly understood. The role of ncRNAs in epilepsy is indispensable for this unknown field. Thus, it is worth for further exploration which might contribute to therapeutic strategies for epilepsy (Table 3).

Despite that the pathogenesis of epilepsy is not clear, a few studies about ncRNA functions had taken new steps. A validation study revealed that miR-346 and miR-331-3p were significantly downregulated in exosomes from the epileptic forebrain (Gitai et al., 2020). The enrichment pathway analysis of these miRNAs showed an overrepresentation of signaling pathways that were linked to molecular mechanisms underlying chronic epilepsy, including GABA-ergic and mTOR (Gitai et al., 2020). Functional studies on these two miRNAs might uncover their roles in the pathophysiology and treatment of temporal lobe epilepsy (TLE). Furthermore, there are still many ncRNAs closely related to synaptic plasticity, neuronal excitability, neuroinflammatory response, and neuronal death, which are essential basic pathological processes for the occurrence of epilepsy, such as miR-146a, miR-128, lncRNA BC1, and circRNA—CDR1as (Gitai et al., 2011; Geng et al., 2016; He et al., 2016). Nevertheless, no definitive experiment has ever demonstrated that these ncRNAs existed in exosomes of epilepsy patients. Thus, the mechanism of exosomal ncRNA-related epilepsy has emerged as a new study field.

### Amyotrophic Lateral Sclerosis (ALS)

Amyotrophic lateral sclerosis (ALS) is a fatal neurodegenerative disease characterized by the progressive loss of motor neurons (MNs), which usually evolves rapidly and results in death due to respiratory failure within 1–5 years after the onset of the disease (Yang et al., 2021). Furthermore, ALS is a catastrophic neurodegenerative disease caused by partial dysfunction and damage to upper MNs in the primary motor cortex; and lower MNs in the brainstem and spinal cord, resulting in the paralysis of voluntary muscles, stiffness, muscle atrophy, fasciculation swallowing, phonation, and respiratory function depression (Wang K. et al., 2021). These findings indicate that MNs die from one unit to neighboring neurons through mechanisms that involve altered intercellular communication between neurons and glial cells (Garden and La Spada, 2012). Therefore, an increasing number of scientists have begun to pay attention to the role of exosomes and their contents as a mode of cell-to-cell communication in the occurrence of ALS (Table 3).

Several altered factors are involved in the pathogenesis of ALS, including immune disorders, mitochondrial dysfunction, oxidative stress, protein aggregates, neurofilament accumulation, and neuroinflammation. To date, there have been many reports related to exosomes, successively demonstrating the crucial role of exosome-mediated ncRNA in the pathogenesis of ALS given the relevance of RNA homeostasis in disease pathogenesis (Gagliardi et al., 2021). For example, microglia released exosomes enriched for miR-146 and miR-155, which were implicated in the neuroinflammatory process affecting ALS progression (Vaz et al., 2019). A similar study demonstrated that exosomal inflammatory-related miRNAs induced persistent NF-κB signal pathway activation in microglial cells, which might result in aggravated microglial neurotoxicity or death toward MNs and neuroinflammation in ALS patients (Frakes et al., 2014; Pinto et al., 2017). Xu and colleagues found a reduction in exosomal miR-27a-3p in ALS patient serum compared to healthy control serum (Xu et al., 2018). Notably, 40%–55% of familial ALS cases are due to pathogenic mutations in disease-related genes, such as SOD1, TARDBP, and C9orf72, which are the most frequently involved (Perrone and Conforti, 2020). Meanwhile, it has been proven that these gene mutations might refer to exosomal ncRNA (Rinchetti et al., 2018; Varcianna et al., 2019; Nishimoto et al., 2021). Unfortunately, the detailed molecular mechanism of ALS is still unclear. Thus, further investigation is needed to elucidate the role of exosomal ncRNAs in ALS, which promotes the development of early and specific diagnostic methods.

### Depression

Mental disorder is a significant concern for healthcare systems worldwide and a cumbersome burden to both individuals and society (Eaton et al., 2008). Depression impacts an estimated 350 million people worldwide, which is a typical neuropsychiatric disease and is associated with genetic factors (Kessler and Bromet, 2013). Kessler with his colleagues analyzed 191 unique miRNAs across 35 human studies, then they provided an insightful understanding that the molecular biology of mental disorders and physiological explanation of psychological changes was possible (Gruzdev et al., 2019). GO and KEGG enrichment analysis indicated that the differential expression of exosomal miRNAs might play an important role in the pathogenesis of depression through the MAPK pathway, Wnt pathway, and mTOR pathway (Fang et al., 2020). Moreover, Xian observed that BV2 microglial cells successfully internalized PC12 neuron cell-derived exosomes as well as transferred miR-9-5p (Xian et al., 2022). miR-9-5p promoted M1 polarization in microglia and led to over releasing of proinflammatory cytokines, such as IL-1β, IL-6, and TNFα. Furthermore, miR-9-5p overexpression suppressed SOCS2 expression and reactivated SOCS2-repressed Janus kinase (JAK)/signal transducer and activator of transcription 3 (STAT3) pathways. From this study they confirmed that adeno-associated virus (AAV)-mediated overexpression of exosomal miR-9-5p polarized microglia toward the M1 phenotype and exacerbated depressive symptoms in chronic unpredictable mild stress mouse mode. Similarly, overexpression of exosomal miR-146a-5p in hippocampal dentate gyrus suppressed neurogenesis and spontaneous discharge of excitatory neurons by directly targeting Kruppel-like factor 4 (KLF4; Fan et al., 2022). Furthermore, miR-146a-5p downregulation reduced adult neurogenesis deficits and depression-like behaviors in rats. Deeper research found that circular RNA ANKS1B acted as a miRNA sponge for miR-146a-5p to mediate post-transcriptional regulation of KLF4 expression. In general, this study indicates that exosomal miR-146a-5p can function as a vital factor to regulate the pathological processes of neurogenesis resulting from depression.

Intriguingly, *in vivo* experiments showed that neuron-derived exosomes decreased the levels of pro-inflammatory cytokines (IL-1β, IL-6, and TNFα; Li et al., 2020). With more detailed research, miR-207 was found to be overexpressed in exosomes and experiments confirmed that exosomal miR-207 directly targeted TLR4 interactor with leucine-rich repeats (Tril) and inhibited NF-κB signaling in astrocytes. In conclusion, exosomal miR-207 could decrease the release of pro-inflammatory cytokines and inhibit the expression of Tril in brain, which provides promising molecular therapies to decrease antidepressant activity.

## Exosomal NcRNA as A Potential Biomarker in The Diagnosis of CNS Diseases

Society has witnessed a skyrocketing increase in the incidence of CNS diseases, such as brain tumors, neurodegenerative diseases (AD, PD, etc.), stroke, epilepsy, MS, and ALS, which have seriously undermined the quality of life and substantially increased economic and societal burdens (Zhang et al., 2021). Thus, early diagnosis and treatment strategies with a good prognosis are needed to reduce the harm to the physical and mental health of individuals and to relieve the financial burden of families and the social medical pressure. At present, medical checkups and imaging studies, such as electroencephalogram, magnetic resonance imaging, and neuropsychological examination, are the gold standard diagnostic methods for patients with CNS disease. While these methods do help some people get diagnosed and treated, the early stages of CNS disease are not visual, and psychiatric symptoms often represent the clinical onset and refractory period of such diseases, thus potentially leading to misdiagnosis, delays in treatment, and worse outcomes (Menculini et al., 2021). Moreover, the neurological test cannot accurately determine the subtype and stage of CNS disease due to the subjectivity of doctors and imperceptible differences between very similar symptoms. Many studies have reported some biomarkers for CNS disease diagnosis, including circulating proteins, enzymes, and circulating DNAs, but these markers also have inconvenient drawbacks. For example, the number is too small to detect, samples are difficult to collect, the sensitivity is inadequate, and the specificity needs to be improved (Olsson et al., 2011; McKeever et al., 2018; Yang T. T. et al., 2018; Lange, 2021). However, as we mentioned above, exosomes contain multifarious functional ncRNAs that may reflect the typical stages and types of CNS diseases (Xia et al., 2019; He et al., 2021). Moreover, exosomal ncRNAs are relatively steadily expressed and are readily accessible in a variety of human biofluid types (Bullock et al., 2015). Furthermore, given the irreplaceable role of exosomal ncRNAs in physiological and pathological processes in CNS diseases, exosomal ncRNAs are an emerging field for clinical diagnosis and have attracted increasing attention (Xu et al., 2018; Liu et al., 2019; Xia et al., 2019; Hornung et al., 2020; Wang and Zhang, 2020; Yu et al., 2021; Table 4).

**Table 4:** The biomarker potential of exosomal ncRNAs in Alzheimer’s diseases, CNS tumors, multiple sclerosis, epilepsy, and amyotrophic lateral sclerosis.

**Diseases**	**Classification of ncRNA**	**ncRNAs**	**Regulation**	**ROC analysis**	**Biomarker potential**	**Source of exosome**	**Reference**
Alzheimer’s disease	miRNA	miR-135a	Up	-	A biomarker for AD early stages diagnosis	Serum, CSF	Liu C. G. et al. (2021)
		miR-193b	Down	-	A blood-based biomarker for MCI and DAT patients	Blood, CSF	Liu et al. (2014)
		miR-34b, miR-29a	Up	AUC = 0.812, sensitivity = 83% (vs. VaD) specificity = 74% (vs. VaD); AUC = 0.832 sensitivity = 63% (vs. VaD) specificity = 96% (vs. VaD)	Biomarkers to discriminate clinically similar neurodegenerative and vascular-related diseases	Serum	Barbagallo et al. (2020)
		miR-16-5p, miR-451a, miR-605-5p	Down	AUC = 0.760 AUC = 0.951 AUC = 0.706	A biomarker for YOAD diagnosis	CSF	McKeever et al. (2018)
		miR-125b-5p	Up	AUC = 0.723			
		miR-384	Up	AUC = 0.991 (vs. PDD) sensitivity = 99.07% (vs. PDD) specificity = 100% (vs. PDD) AUC = 0.991 (vs. VaD) sensitivity = 99.10% (vs. VaD) specificity = 100% (vs. VaD)	A biomarker for AD diagnosis and discrimination between AD, VaD, and PDD	Serum	Yang T. T. et al. (2018)
		miR-135a	Up	AUC = 0.598 (vs. PDD) sensitivity = 75.70% (vs. PDD) specificity = 46.67% (vs. PDD); AUC = 0.721 (vs. VaD) sensitivity = 89.70% (vs. VaD) specificity = 55% (vs. VaD)			
	lncRNA	Bace1-AS	Up	AUC = 0.761 sensitivity = 87.5% specificity = 61.3%	A novel biomarker for AD diagnosis	Plasma	Wang D. et al. (2020)
	circRNA	KIAA1586	Up	-	A potential biomarker for AD diagnosis	Blood	Zhang et al. (2019)
	piRNA	piR_019949	Up	AUC = 0.96	Predict conversion from MCI to AD dementia	CSF	Jain et al. (2019)
		piR_020364		AUC = 0.89	A biomarker for classifying AD dementia patients		
		piR_019324	Down				
CNS tumor	miRNA	miR-454-3p	Down	AUC = 0.866 sensitivity = 79.17% specificity = 91.67%	An exosomal biomarker for glioma diagnosis and prognosis	Serum	Shao et al. (2019)
		miR-21	Up	AUC = 0.927 (vs. health) AUC = 0.872 (grade III/VI vs. II) AUC = 0.751 (grade VI vs. II)	A biomarker for glioma diagnosis, prognosis and different grade	CSF	Shi et al. (2015)
		RNU6	Up	AUC = 0.852	A biomarker for GBM diagnosis	Serum	Manterola et al. (2014)
		miR-574-3p	Up	AUC = 0.738			
		miR-320	Up	AUC = 0.719			
	lncRNA	HOTAIR	Up	AUC = 0.913 sensitivity = 86.1% specificity = 87.5%	A biomarker for GBM diagnosis	Serum	Tan et al. (2018)
		HOTAIR	Up	-	Promising prognostic predictors for GBM	Serum	Wang Z. et al. (2021)
		SOX21-AS1	Down				
		STEAP3-AS1	Up				
	circRNA	circNFIX	Up	AUC = 0.885	A biomarker for glioma diagnosis and prognosis	Serum	Ding et al. (2020)
		circHIPK3	Up	-	A potential biomarker for the TMZ-resistant glioma diagnosis	Serum	Yin and Cui (2021)
		circMMP1	Up	AUC = 0.8144	A biomarker for glioma diagnosis and prognosis	Serum	Yin and Liu (2020)
Multiple sclerosis	miRNA	miR-15b-5p	Up	AUC = 0.740	Biomarkers for RRMS diagnosis		Ebrahimkhani et al. (2017)
		miR-122-5p	Down	AUC = 0.878	Biomarkers for RRMS diagnosis	Serum	Selmaj et al. (2017)
		hsa-miR-196b-5p	Down	AUC = 0.866			
		miR-19b, miR-25 and miR-92a	Up	-	Potential exosomal biomarkers for MS diagnosis	Plasma	Kimura et al. (2018)
		miR-326	Up	-	A biomarker for MS diagnosis	Serum	Azimi et al. (2019)
		miR-22-3p, miR-660-5p	Down	-	Biomarkers for MS diagnosis and the response after IFN-b therapy	Serum	Manna et al. (2018)
Epilepsy	miRNA	miR-328-3p	Up	AUC = 0.63 (vs. TLE) AUC = 0.90 (EAS vs. EBS)	A novel biomarker for epilepsy diagnosis and different subtypes	Plasma	Raoof et al. (2018)
		miR-8071	Down	AUC = 0.932 sensitivity = 83.33% specificity = 96.67%	A novel biomarker for TLE-HS diagnosis	Plasma	Yan et al. (2017)
		miR-451a, miR-21-5p, miR-19b-3p	Up Down Up	AUC = 0.80	Biomarkers for TLE diagnosis	CSF	Raoof et al. (2017)
ALS	miRNA	miR-27a-3p	Down	-	Biomarkers for ALS diagnosis	Serum	Xu et al. (2018)
		miR-124-3p	Down	-	A disease stage indicator in ALS	CSF	Yelick et al. (2020)
		miR-199a-3p miR-151a-5p	Up	-	Biomarkers for ALS/MND diagnosis	plasma	Banack et al. (2020)

These factors make exosomal ncRNAs the most promising biomarkers of CNS diseases. For example, in 2021, Liu observed increased levels of ABCA1-labeled exosomal miRNA-135a in the CSF of the AD group compared to those of the control group (*P* < 0.05) and significantly increased levels in mild cognitive impairment (MCI) and dementia of Alzheimer-type (DAT) patient groups compared to those of the control group (*P* < 0.05; Liu C. G. et al., 2021). Another study proved that 20 exosomal miRNAs showed distinct differences in the AD group in preliminary screening, and seven of these exosomal miRNAs (miR-342-3p, miR-141-3p, miR-342-5p, miR-23b-3p, miR-24-3p, miR-125b-5p, and miR-152-3p) were sufficient to predict the status of an individual sample with 83%–89% accuracy (Lugli et al., 2015). Additionally, manifold other studies have certified exosomal ncRNAs as a diagnostic tool for CNS diseases (Table 5).

**Table 5:** The biomarker potential of exosomal ncRNAs in Parkinson’s diseases and stroke.

**Diseases**	**Classification of ncRNA**	**ncRNAs**	**Regulation**	**ROC analysis**	**Biomarker potential**	**Source of exosome**	**Reference**
Parkinson disease	miRNA	miR-34a-5p	Up	AUC = 0.740	A potential biomarker for PD diagnosis	Plasma	Grossi et al. (2021)
		miR-331-5p	Up	AUC = 0.849	Biomarkers for PD diagnosis	Plasma	Yao et al. (2018)
		miR-505	Down	AUC = 0.898			
		miR-19b	Down	AUC = 0.753 sensitivity = 68.8% specificity = 77.5%	Biomarkers for PD diagnosis	Serum	Cao et al. (2017)
		miR-24	Up	AUC = 0.908 sensitivity = 81.7% specificity = 85.0%			
		miR-195	Up	AUC = 0.697 sensitivity = 82.6% specificity = 55.0%			
		miR-1	Down	AUC = 0.920	Reliable biomarkers for PD diagnosis	CSF	Gui et al. (2015)
		miR-409-3p	Up	AUC = 0.970			
		miR-19b-3p	Down	AUC = 0.705			
		miR-10a-5p	Up	AUC = 0.900			
	lncRNA	RP11-462G22.1, PCA3	Up	-		CSF	Gui et al. (2015)
		POU3F3	Up	AUC = 0.763 sensitivity = 68% specificity = 72%	A biomarker is significantly correlated with PD severity and for PD diagnosis	Plasma	Zou et al. (2020) and Kuo et al. (2021)
		AK127687, AX747125, SNCA-AS1, UCHL1-AS1, PINK1AS1, MAPT-AS1	Down	-	Representing novel targets for PD diagnosis	Blood	Elkouris et al. (2019)
Stroke	miRNA	miR-134	Up	AUC = 0.834 sensitivity = 75.3% specificity = 72.8%	A novel biomarker for the diagnosis and prognosis of stroke	Serum	Zhou et al. (2018)
		miR-223	Up	-	A potential biomarker correlated with stroke severity and stroke diagnosis	Blood	Chen et al. (2017b)
		miR-21-5p	Up	AUC = 0.714 (vs. SIS) AUC = 0.734 (vs. RIS)	Biomarkers for different phases and diagnoses of stroke	Plasma	Wang et al. (2018)
		miR-30a-5p	Up	AUC = 0.826 (vs. HIS)			
		miR-9 miR-124	Up	AUC = 0.8026 AUC = 0.6976	Promising biomarkers for the ischemic stroke diagnosis and predict the ischemic damage	Serum	Ji et al. (2016)

As stated, lncRNAs play important roles in the pathological development of various CNS diseases accompanied by upregulation or downregulation of expression, suggesting that lncRNAs could be biomarkers for CNS disease diagnosis and meet additional biological and statistical criteria. In 2020, Zou et al. (2020) found highly upregulated expression of linc-POU3F3 in plasma L1CAM exosomes of PD patients compared with healthy controls (Zou et al., 2020). Moreover, a recent study published by Wang Q. et al. (2020) revealed 15 PD-relevant exosomal lncRNAs with upregulated expression and 24 exosomal lncRNAs with downregulated expression. For example, among those differentially expressed exosomal lncRNAs, MSTRG.242001.1 and MSTRG.169261.1 were highly expressed in PD patients. Hence, exosomal linc-POU3F3, lncRNA-MSTRG.242001.1, and lncRNA-MSTRG.169261.1 might be potential biomarkers to improve the diagnostic efficiency of PD.

Except for miRNAs and lncRNAs, which are classical noncoding RNAs, there are still plenty of other exosomal ncRNAs that can serve as promising biomarkers. For instance, the team of Jain et al. defined a combined signature consisting of three exosomal miRNAs (miR-27a-3p, miR-30a-5p, and miR-34c) and three exosomal piRNAs (piR_019324, piR_019949, and piR_020364) that were appropriate to diagnose AD with an AUC of 0.83 (Jain et al., 2019).

Not only could exosomal ncRNAs be used as biomarkers to diagnose complex CNS diseases, but among these ex-ncRNAs, they also favor distinguishing analogous neurodegenerative diseases and judging progression and subtype. For example, serum exosomal miR-193b was downregulated, while miR-135a and miR-384 were upregulated in AD patients compared to control groups (Yang T. T. et al., 2018). Among these three miRNAs, the expression of serum exosomal miR-384 was higher in AD patients than in vascular dementia (VD) and Parkinson’s disease with dementia (PDD) patients (Yang T. T. et al., 2018). This result indicated that miR-384 appeared to be a biomarker for discriminating AD, VD, and PDD. It is worth setting forth that a reduced content of exosomal miR-16-5p in young-onset AD (YOAD, <65 years), but not in late-onset AD (LOAD, >65 years), was detected in CSF-derived exosome samples compared to controls (McKeever et al., 2018). Interestingly, with further study, Gui and colleagues analyzed several miRNAs, mRNA transcripts, and lncRNAs present in CSF exosomes from both PD and AD patients (Gui et al., 2015). In particular, miR-10a-5p, let-7c-3p, miR-153, and miR-409-3p were obviously upregulated in PD CSF exosomes compared with AD and control exosomes. However, exosomal miR-1 and exosomal miR-19b-3p were downregulated (Gui et al., 2015; Leggio et al., 2017). These studies indicated the potential of a specific subset of exosomal miRNAs to distinguish between AD and PD. In conclusion, existing studies have verified the feasibility of large-scale clinical applications of exosomal ncRNAs as biomarkers for CNS disease diagnosis and the differentiation of disease stages and types (Chen J. J. et al., 2019; He et al., 2021).

Above all, developing performance accuracy methods for the detection, differentiation, and prognosis of CNS diseases will be clinically meaningful. These above mentioned studies suggest that exosomal ncRNAs have shown outstanding potential as novel tools for CNS disease diagnosis, and their clinical applications deserve further investigation.

## Exosomal NcRNA as A Potential Therapeutic Strategy for CNS Diseases

**Figure 3:**
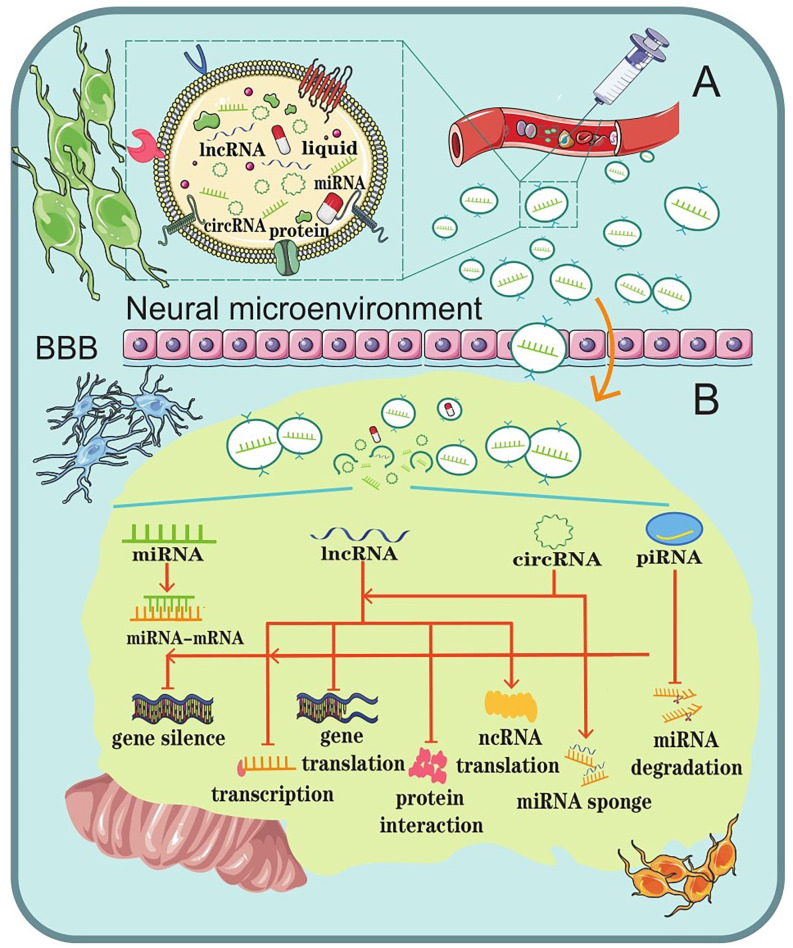
Schematic representation of the role of exosomal ncRNAs as a potential therapeutic strategy for CNS diseases. **(A)** Liposoluble exosomes can cross the BBB easily. They are intended as natural nanoscale vesicles; therefore, they can be engineered to delete vehicles that contain specific drugs or biological ncRNAs. **(B)** The biological functions of exosomal ncRNAs in the brain microenvironment.

Once a CNS disease occurs, it usually cannot be repaired by the body’s internal repair system. Instead, it usually needs to be treated with advanced medical care. Unfortunately, CNS disease seems to be increasing around the world. However, this has also brought about an increase in research and treatments, though they have their own defects more or less. A major problem to overcome in CNS disease treatment is trying to overcome the problem of BBB. The BBB protects the brain from many types of diseases but also makes it difficult to create a drug treatment that targets the specific regions in brain (Dong, 2018). Because of the biocompatibility of exosomes and the singular function of ncRNAs, scientists in mounting numbers have been exploring exosomal ncRNAs as a promising strategy for CNS disease therapy (Li et al., 2018; Iranifar et al., 2019; Xia et al., 2019; Wu and Kuo, 2020; Dolati et al., 2021; Mattingly et al., 2021; Figure 3).

Exosomes, which are secreted by the majority of cells, are involved in numerous neurophysiological functions, such as growth, differentiation, proliferation, and cell death (Lopez-Verrilli and Court, 2013). Since liposoluble exosomes can easily cross the BBB, they might be used as natural nanoscale drug delivery vesicles (Yu et al., 2020). In addition, since exosomes are part of the body’s intercellular carriers of patients, using them to transfer drugs into the recipient cells would probably not evoke the immune system (Iranifar et al., 2019).

This dual advantage could potentially make exosomes a strategy with safety and effectiveness for the treatment of CNS diseases. For example, *in vitro* and *in vivo* studies have demonstrated that dopamine can be successfully loaded into exosomes and delivered to the striatum and substantia nigra. This had a better therapeutic effect in a PD mouse model and showed less toxicity than free dopamine by intravenous systemic administration (Qu et al., 2018). Similarly, many studies have reported the successful loading of exosomes with catalase, dopamine, catalase mRNA, and small interfering RNA (Alvarez-Erviti et al., 2011; Luo et al., 2020). The findings disclosed the therapeutic potential of exosome-mediated targeting for CNS disease treatment and showed striking therapeutic effects.

In addition to exosomes themselves, the ncRNAs in the exosomes are regarded as another restorative option. This could exert their effects by influencing various cellular and molecular pathways involved in CNS diseases. Emerging evidence suggests that specific exosomes induce a pro-neurogenesis effect, which is attributed to histone deacetylase 6 inhibition *via* the transfer of exosomal miR-26a. Thus, Ling et al. (2020) indicated that exosomal ncRNAs can be used as a novel and promising strategy for brain ischemia. *In vivo* tumorigenesis experiments performed by Chai showed that exosomal lnc-ROR1-AS1 promoted glioma development by inhibiting the miR-4686 axis, indicating that upregulating the expression of exosomal lnc-ROR1-AS1 could alleviate the progression of glioma (Chai et al., 2020). Therefore, it seems that exosomal ncRNAs could be used as potential therapeutic candidates in CNS disease treatment.

In conclusion, ongoing studies have proposed that exosomes could be applied as a promising therapeutic delivery system by targeting their cargos to recipient cells, and ncRNAs in endogenous cell-derived exosomes may have the potential to adjust neurogenesis to treat various CNS diseases. However, exosomes and the ncRNAs they contain, are commonly limited in scale (Liu et al., 2022a, b). We still do not have comprehensive methods to investigate this (Liu Y. et al., 2021). This wealth of raw information requires time-consuming experiments to pinpoint the benefits and harm of less conserved ncRNAs in the pathogenesis of CNS diseases (Xia et al., 2019). Further study of the association between other biomolecules and exosome needs to be considered in the future (Ehrlich et al., 2016; Liu et al., 2022c, d). Thus, a massive amount of further study is needed before these therapeutic approaches will be available for clinical application.

## Conclusions and Perspectives

CNS diseases persist to be one of the most demanding burdens on medical and health care services as well as being one of the most complex of diseases. Moreover, our understanding of their pathologic mechanisms is relatively poor. Their pathogenesis has long been a source of confusion, which is the reason for the difficulty in early diagnosis and the poor prognosis of CNS diseases. Exosomes constitute a novel form of cell-to-cell interaction and communication with manifold components. Current studies indicate that exosomes in the microenvironment have crucial impacts on both interneuron and neuron-glia cells by transferring their contents, such as proteins, lipids, and ncRNAs. Moreover, exosomal ncRNAs, including miRNAs, lncRNAs, circRNAs, and piRNAs, regulate physiological functions and play vital roles in sustaining CNS homeostasis. Convincing discoveries present the potential value of exosomal ncRNAs as diagnostic tools and therapeutic strategies in clinical applications due to their ability to cross the biological barrier and deliver cargo to recipient cells.

However, as mentioned above, the lack of perception of the mechanisms of CNS diseases and approaches for selecting disease-specific exosomal ncRNAs and limitations in scale are nonnegligible barriers to their application in clinical settings. The large amounts of exosomal ncRNA information available require time-consuming experiments to pinpoint the valuable functions of these ncRNAs in the pathogenesis of CNS diseases. This will undoubtedly require large amounts of manpower, material and financial resources, and will require the screening of a large clinical sample size. Furthermore, it is worth pointing out that exosomal ncRNAs regulated by pathogenic genes are heterogeneous. Thus, further extensive research needs to distinguish the differences between the initial heterogeneity of the cell response and minor adjustments of pathological processes.

Meanwhile, pioneering studies that focus on exosomal ncRNAs in the CNS have produced results that are lacking precision and sometimes even contradict each other. Additionally, these studies ignore the fact that subtly monitoring and commanding exosomes to recipient regions in the application of exosomal ncRNA as therapeutic “drugs” is still a challenging prospect. In conclusion, based on the present detailed insights into the current state of ncRNAs, a better understanding of the expression patterns and pathological roles of exosomal ncRNAs in CNS diseases is needed. The recruitment of exosomal miRNAs as promising biomarkers in the diagnosis and therapeutic strategies in the treatment of CNS diseases is a near possibility.

## Author Contributions

Z-YW, Z-JW, and YZ searched the literature. H-MX and Y-FZ provided inspiration and guidance for writing. Z-YW and Z-JW wrote the manuscript and prepared all the figures and tables. All authors contributed to the article and approved the submitted version.

## Funding

This work was supported by the National Natural Science Foundation of China (22006084; 81660527), Ningxia Natural Science Foundation (2022AAC05027), and the Qingdao Applied Basic Research Project (19-6-2-49-cg).

## Abbreviations

CSF: cerebrospinal fluid
VaD: Vascular dementia
YOAD: young-onset Alzheimer’s disease
PDD: Parkinson’s disease with dementia
TMZ: temozolomide
SIS: subacute phase ischemic stroke
RIS: recovery phase ischemic stroke
HIS: hyperacute phase ischemic stroke
EAS: epilepsy after-seizure sample
EBS: epilepsy baseline sample
MND: motor neuron disease
TLR-HS: temporal lobe epilepsy with hippocampal sclerosis.
